# Effect of methanol extracts of *Cnidium officinale* Makino and *Capsella bursa-pastoris* on the apoptosis of HSC-2 human oral cancer cells

**DOI:** 10.3892/etm.2012.871

**Published:** 2012-12-21

**Authors:** KYUNG-EUN LEE, JI-AE SHIN, IN-SUN HONG, NAM-PYO CHO, SUNG-DAE CHO

**Affiliations:** 1 Department of Oral Medicine, Chonbuk National University; Jeonju 561-756;; 2 Department of Oral Pathology, School of Dentistry, Institute of Oral Bioscience, BK21 Project, Chonbuk National University, Jeonju 561-756;; 3 Department of Veterinary Public Health, Laboratory of Stem Cell and Tumor Biology, Seoul National University, Seoul 151-742, Republic of Korea

**Keywords:** *Cnidium officinale* Makino, *Capsella bursa-pastoris*, specificity protein 1, oral cancer, apoptosis

## Abstract

*Cnidium officinale* Makino and *Capsella bursa-pastoris* are used as traditional herbs with diverse medicinal effects, including the inhibition of inflammation, reduction of blood pressure and as diuretics, however, the anti-cancer effects of *C. officinale* Makino and *C. bursa-pastoris* are poorly defined. The aims of this study were to evaluate the effects of methanol extracts of *C. officinale* Makino (MECO) and methanol extracts of *C. bursa-pastoris* (MECB) on the cell growth and apoptosis of HSC-2 human oral cancer cells. MECO and MECB caused growth inhibition and the induction of apoptosis in a concentration-dependent manner in HSC-2 cells. A marked reduction in specificity protein 1 (Sp1) expression following treatment with MECO or MECB was also observed. The downregulation of Sp1 by siRNA resulted in growth inhibition and a reduction of total poly (ADP-ribose) polymerase (PARP) expression. In addition, MECO significantly increased Bax expression levels and MECB increased Bak expression levels and decreased Mcl-1 expression levels. These results suggest that MECO and MECB inhibit cell growth and induce apoptosis via the Sp1 protein, indicating that MECO and MECB are useful bioactive materials and attractive drug candidates for oral cancer.

## Introduction

Naturally occurring compounds have attracted considerable attention as cancer chemopreventive agents due to their beneficial effects on human health and their potent anticancer effects (1). Certain naturally occurring compounds have been reported to effectively suppress cell proliferation and tumor progression in *in vivo* and *in vitro* experimental models of cancer by inducing apoptosis (2). In particular, naturally occurring compounds derived from plant sources, including curcumin, polyphenols, betulinic acid and ellagic acid, have been studied in various models as modulators of proliferation, angiogenesis, apoptosis and inflammation (3). It has also been demonstrated that the antitumor activities of naturally occurring compounds are associated with the regulation of numerous molecular targets, including p53, VEGF, STAT3, MAPK and PI3K/AKT signaling pathways (1,3). Therefore, it is important to understand the antitumor effects and molecular mechanisms of naturally occurring compounds for chemoprevention and chemotherapy.

Specificity protein 1 (Sp1) is a transcription factor which binds GC/GT-rich promoter elements via three Cys_2_His_2_-type zinc fingers and plays key roles in tumorigenesis (4,5). Sp1 regulates several cancer associated genes associated with the cell cycle, proliferation, differentiation and apoptosis (6). In addition, Sp1 is overexpressed in several cancers and is closely correlated with the prognosis of patients (7–9). Notably, naturally occurring compounds, such as curcumin and betulinic acid, have been reported to suppress tumor growth via the downregulation of Sp1 expression in prostate and bladder cancer cells (10,11). Isorhapontigenin has also demonstrated an anticancer effect by inducing apoptosis through the down-regulation of the Sp1/XIAP pathway (12). Therefore, the downregulation of Sp1 by naturally occurring compounds may be a potential chemopreventive and chemotherapeutic strategy for cancer.

The present study demonstrates that methanol extracts of *C. officinale* Makino (MECO) and *C. bursa-pastoris* (MECB) decrease cell growth and induce apoptosis via the downregulation of Sp1 in HSC-2 human oral cancer cells.

## Materials and methods

### Chemicals and antibodies

MECO and MECB were provided by Professor Ki-Han Kwon (Kwangju University, Kwangju, Korea). The DC Protein Assay kit was acquired from Bio-Rad Laboratories Inc., (Madison, WI, USA). Sp1 and actin antibodies were obtained from Santa Cruz Biotechnology (Santa Cruz, CA, USA). Poly (ADP-ribose) polymerase (PARP) antibody was provided by BD Pharmingen™ (San Jose, CA, USA). Antibodies against Bak, Bax, Bcl-xL and Mcl-1 were supplied by Cell Signaling Technology (Charlottesville, VA, USA).

### Cell culture and chemical treatment

HSC-2 human oral cancer cells were provided by Hokkaido University (Hokkaido, Japan). Cells were maintained in DMEM supplemented with 10% fetal bovine serum and 1% penicillin/streptomycin at 37°C in a 5% CO_2_ incubator. The cells were treated with DMSO or various concentrations (300, 600 and 900 *μ*g/ml) of MECO or MECB for 24 or 48 h.

### MTS assay

The effects of MECO and MECB on cell growth were investigated using the CellTiter 96® Aqueous One Solution Cell Proliferation Assay kit (Promega, Madison, WI, USA). Cells were seeded in 96-well plates and treated with MECO or MECB for 24 or 48 h. MTS solution was added to each well and incubated for 2 h at 37°C. The absorbance was measured using an ELISA microplate reader (Bio-Tek Instruments, Inc., Madison, WI, USA) at 490 and 690 nm (as a blank control).

### Detection of nuclear morphological changes

The effects of MECO and MECB on nuclear morphological change were confirmed using the fluorescent nuclear dye, DAPI (Sigma, St. Louis, MO, USA). HSC-2 cells were seeded and treated with MECO or MECB for 48 h. The cells were harvested by trypsinization, resuspended in PBS and then fixed in 100% methanol at room temperature for 10 min. The cells were deposited on slides and stained with DAPI solution (2 *μ*g/ml). The cell morphological change was observed using a fluorescence microscope equipped with a suitable filter for the DAPI fluorescent dye.

### Western blot analysis

The protein concentration of the supernatant was determined using the DC Protein Assay kit. The samples containing equal amounts of protein were resolved by SDS-PAGE and transferred to Immun-Blot PVDF membranes (Bio-Rad Laboratories, Hercules, CA, USA). The membranes were blocked with 5% skimmed milk in TBST at room temperature for 2 h and probed overnight at 4°C with various primary antibodies. They were then incubated with HRP-conjugated secondary antibodies. After 2 h, the membranes were washed and detected using the ECL Western Blotting Luminol reagent (Santa Cruz Biotechnology).

### RNA interference

ON-TARGETplus SMARTpool siRNA sequences targeting Sp1 and non-targeting control were supplied by Dharmacon Research (Lafayette, CO, USA). The HSC-2 cells were seeded in 6-well plates and transiently transfected with 25 nM siRNA using a DharmaFECT2 transfection reagent (Thermo Scientific, Lafayette, CO, USA). After 48 h, transfected cells were harvested and examined by trypan blue exclusion assay and western blot analysis.

### Trypan blue exclusion assay

The HSC-2 cells were transfected with 25 nM siRNA for 48 h and the number of viable cells was counted using a hemocytometer with trypan blue (0.4%). The result was expressed as the mean ± standard deviation.

### Statistical analysis

A Student’s t-test was used to determine the significance of differences between the control and treatment groups. P<0.05 was considered to indicate a statistically significant result.

## Results

### MECO and MECB inhibit cell growth and induce apoptosis in HSC-2 human oral cancer cells

**Figure 1.**
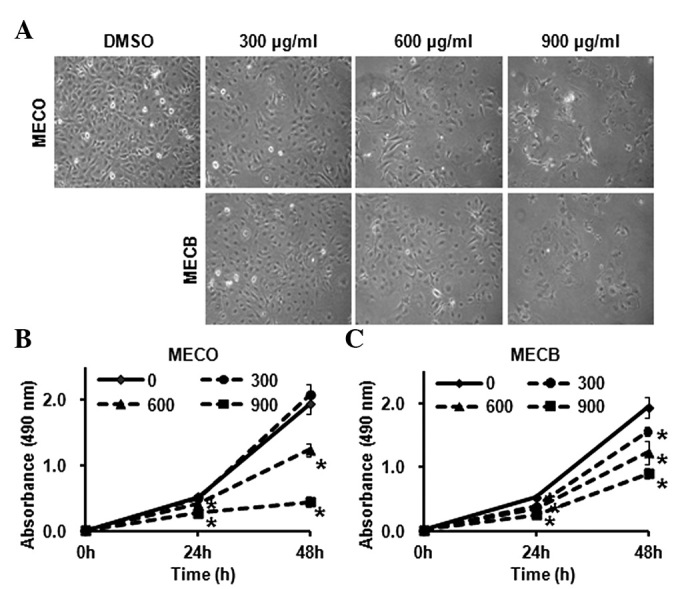
Growth inhibitory effects of MECO and MECB in HSC-2 cells. (A) HSC-2 cells were treated with DMSO or various concentrations (300, 600 and 900 *μ*g/ml) of MECO or MECB for 24 or 48 h. The cell morphology was observed by optical microscope after 48 h, magnification, ×200. (B) The effects of MECO and MECB on cell growth were examined using an MTS assay. The results of the inhibitory rates of cell growth were determined from the absorbance at 490 nm. The mean absorbance values are representative of the relative number of viable adherent cells. ^*^P<0.05 compared with the DMSO-treated group. MECO, methanol extracts of *C. officinale* Makino; MECB, methanol extracts of *C. bursa-pastoris.*

**Figure 2.**
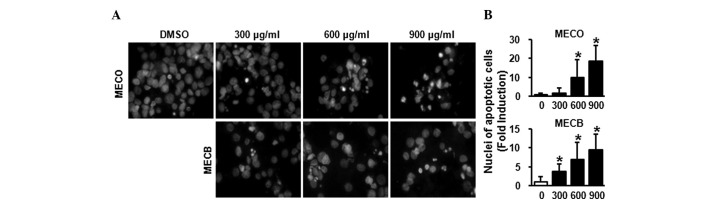
Apoptotic effects of MECO and MECB on HSC-2 cells. (A) HSC-2 cells were treated with DMSO or various concentrations (300, 600 and 900 *μ*g/ml) of MECO or MECB for 48 h. The apoptotic effects of MECO and MECB were determined by DAPI staining. The DAPI-stained cells were observed by fluorescence microscopy, magnification, ×400. (B) The apoptotic cells were counted and expressed as the mean ± standard deviation. ^*^P<0.05 compared with the DMSO-treated group. MECO, methanol extracts of *C. officinale* Makino; MECB, methanol extracts of *C. bursa-pastoris.*

To investigate the anticancer effects of MECO and MECB on HSC-2 human oral cancer cells, we assessed the growth inhibitory effects of MECO and MECB. Cells were treated with DMSO or various concentrations (300, 600 and 900 *μ*g/ml) of MECO or MECB for 24 or 48 h. Morphological changes of the MECO- and MECB-treated cells were observed under an optical microscope after 48 h. As shown in Fig. 1A, a number of MECO- and MECB-treated cells were detached in the medium in a concentration-dependent manner. The effects of MECO and MECB on cell viability were examined using an MTS assay. The results showed that MECO and MECB significantly decreased cell viability in HSC-2 cells (Fig. 1B and C). We then evaluated whether the growth inhibitory effects of MECO and MECB were associated with apoptotic effects using DAPI staining. As shown in Fig. 2A and B, cells treated with MECO or MECB for 48 h exhibited nuclear fragmentation and chromatin condensation in a concentration-dependent manner. These results demonstrate that MECO and MECB inhibited cell growth and induced apoptosis in HSC-2 human oral cancer cells.

### Downregulation of Sp1 by MECO and MECB correlates with the regulation of several Bcl-2 family proteins

**Figure 3.**
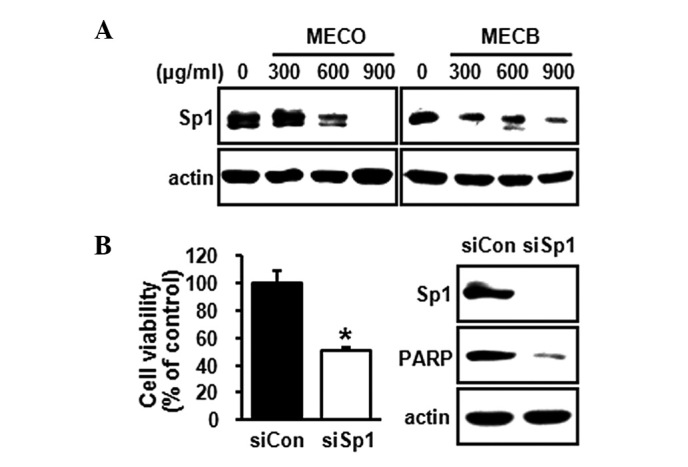
Effects of MECO and MECB on Sp1 expression. (A) HSC-2 cells were treated with DMSO or various concentrations (300, 600 and 900 *μ*g/ml) of MECO or MECB for 48 h. The effects of MECO and MECB on Sp1 expression were analyzed by western blot analysis. (B) HSC-2 cells were transfected with siRNA (siCon or siSp1) for 48 h. The cell viability was estimated by trypan blue exclusion assay and whole-cell lysates were analyzed by western blot analysis using antibodies against Sp1, PARP and actin. MECO, methanol extracts of *C. officinale* Makino; MECB, methanol extracts of *C. bursa-pastoris*; Sp1, specificity protein 1; PARP, poly (ADP-ribose) polymerase.

**Figure 4.**
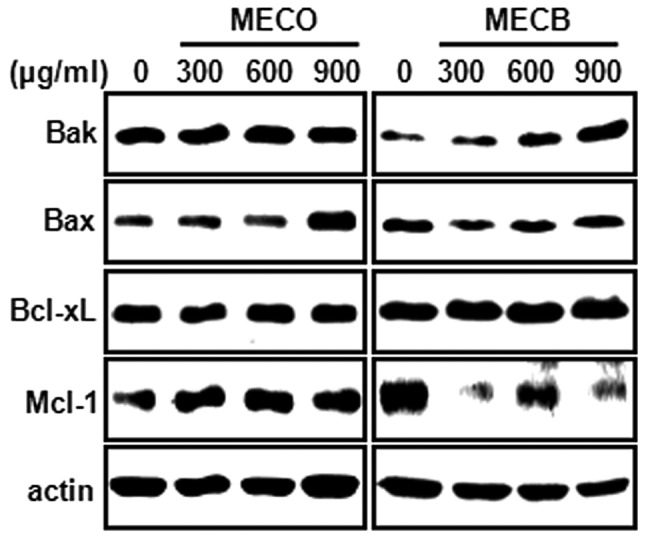
Effects of MECO and MECB on expression of Bcl-2 family proteins. HSC-2 cells were treated with DMSO or various concentrations (300, 600 and 900 *μ*g/ml) of MECO or MECB for 48 h. The expression of Bcl-2 family proteins was detected by western blot analysis using antibodies against Bak, Bax, Bcl-xL and Mcl-1. MECO, methanol extracts of *C. officinale* Makino; MECB, methanol extracts of *C. bursa-pastoris.*

A previous study has suggested that the downregulation of Sp1 inhibits malignant transformation via the induction of apoptosis (13). Since Sp1 is highly expressed in oral tumor tissues compared with normal oral tissues (14), we investigated whether MECO and MECB affect Sp1 expression in HSC-2 cells. The results showed that MECO and MECB decreased Sp1 expression (Fig. 3A). To confirm that the downregulation of Sp1 was associated with the induction of apoptosis, we knocked down the expression of Sp1 in the HSC-2 cells using siRNA technology. As shown in Fig. 3B, the knockdown of Sp1 markedly decreased cell growth and total PARP expression, indicating that downregulation of Sp1 was sufficient to inhibit cell growth and induce apoptosis. To further investigate the molecular mechanism of MECO- and MECB-induced apoptosis, we examined whether MECO and MECB regulated the expression of Bcl-2 family proteins. Fig. 4 shows that MECO caused a marked increase in Bax expression levels. MECB increased Bak expression and decreased Mcl-1 expression. These results indicate that MECO and MECB may induce apoptosis through the regulation of several Bcl-2 family proteins.

## Discussion

Since Sp1 is a critical transcription factor which regulates several cancer associated genes associated with cell survival, proliferation and angiogenesis, the abnormal expression or increased binding activity of Sp1 may contribute to tumor development and progression. In a previous study, the abnormally activated Sp1 expression was shown to represent a potential risk for poor prognosis and directly caused gastric cancer progression (15). The expression of Sp1 in breast cancer tissues is positively associated with TNM stage, tumor invasion and lymph node metastasis (16). In addition, the overexpression of Sp1 is involved in the malignant transformation of human fibroblasts (13). Previous studies have also evaluated different approaches for targeting Sp1, including Sp1 ribozyme, siRNA and dominant negative mutant, in experimental models. The downregulation of Sp1 by Sp1 ribozyme correlated with increased apoptosis (13), and the silencing of Sp1 by siRNA suppressed invasion in human glioma cells (17). The dominant negative mutant of Sp1 demonstrated a growth inhibitory effect in cervical cancer cell lines (18). Therefore, it is likely that Sp1 is an important target for cancer therapy.

A recent study in our laboratory has shown that Sp1 is over-expressed in oral cancer tissues compared with normal oral tissues (14). Notably, several naturally occurring compounds decreased the cell growth of oral cancer cells, and exhibited apoptotic activity through the decreased expression of Sp1 and regulation of its downstream target proteins (14,19,20). These findings demonstrate that certain naturally occurring compounds regulate Sp1 expression to inhibit cell growth and induce apoptosis in oral cancer.

In the present study, we examined whether MECO and MECB inhibit cell growth and induce apoptosis through the downregulation of Sp1 in oral cancer cells. We observed that MECO and MECB significantly decreased cell growth and induced apoptosis, which was caused by Sp1 downregulation.

The Bcl-2 family of proteins have emerged as important regulators in mitochondria-mediated apoptosis due to protein-protein interactions between pro- and anti-apoptotic proteins (21). In particular, it is noteworthy that promoters of Bcl-2 family genes such as Mcl-1 and Bax contain Sp1 binding sites (14,22). Therefore, we hypothesized that the down-regulation of Sp1 by MECO and MECB may affect pro- or anti-apoptotic proteins. We observed that MECO significantly increased Bax expression, but did not change other Bcl-2 family proteins. MECB markedly increased Bak expression and decreased Mcl-1 expression. Although the effects of MECO and MECB on several Bcl-2 family proteins due to the downregulation of Sp1 are unclear, the regulation of Bcl-2 family proteins by MECO and MECB may be due, in part, to the effect of MECO and MECB on Sp1 expression.

In conclusion, the results of the present study indicate that MECO and MECB treatment inhibited cell growth and induced apoptosis via the downregulation of Sp1 in HSC-2 human oral cancer cells. These effects involved the regulation of Bcl-2 family proteins, including Bax, Bak and Mcl-1. Therefore, we provide experimental evidence to indicate that MECO and MECB may be attractive anticancer drug candidates targeting Sp1 in oral cancers.
